# Retraction Note: Antimalarial efficacy of *Pongamia pinnata* (L) Pierre against *Plasmodium falciparum* (3D7 strain) and *Plasmodium berghei* (ANKA)

**DOI:** 10.1186/s12906-021-03312-3

**Published:** 2021-05-10

**Authors:** P. V. V. Satish, K. Sunita

**Affiliations:** Department of Zoology and Aquaculture, Acharya Nagarjuna University, Nagarjuna Nagar, Guntur district, Andhra Pradesh 522510 India

**Retraction to: BMC Complement Altern Med 17, 458 (2017)**

**https://doi.org/10.1186/s12906-017-1958-y**

The Editor in Chief has retracted this article [1] because concerns have been raised regarding two figures presented here. Specifically:

- In Fig. 2 it appears that ‘Control Positive’ and ‘Leaf’ show the same image and that ‘Bark’ and ‘Root’ show the same image
- In Fig. 5 it appears that ‘CH Leaves’ and ‘CH Bark’ show the same image, albeit rotated
- In Fig. 5 it appears that ‘CH Flowers’ and ‘EA Bark’ show the same image, albeit rotated
- In Fig. 5 it appears that ‘HE Bark’ and ‘EA Roots’ show the same image, albeit rotated
- In Fig. 5 it appears that ‘EA Flowers’ and ‘HE Roots’ shows the same image

The authors provided additional data, but were not able to show original images for these figures. The Editor-in-Chief therefore no longer has confidence in the integrity of the data in this article.

Both authors, P.V.V. Satish and K. Sunita have not explicitly stated whether they agree to this retraction notice.

## References

[1] Satish P, Sunita K (2017). Antimalarial efficacy of *Pongamia pinnata* (L) Pierre against *Plasmodium falciparum* (3D7 strain) and *Plasmodium berghei* (ANKA). BMC Complement Altern Med.

